# The association between mental health literacy and positive coping strategies in junior high school students: evidence from a moderated mediation model

**DOI:** 10.3389/fpsyg.2026.1895351

**Published:** 2026-08-24

**Authors:** Chen Chen, Lishuang Zhu, Yang Zhao, Xiaoming Liu

**Affiliations:** 1 Faculty of Psychology, Shinawatra University, Bangkok, Thailand; 2 Department of Psychology, School of Education Science, Jiamusi University, Jiamusi, Heilongjiang, China; 3 Fujin No. 4 Middle School, Jiamusi, Heilongjiang, China; 4 Faculty of Psychology, Northeast Normal University, Changchun, Jilin, China

**Keywords:** junior high school students, mental health literacy, perceived teacher support, positive coping strategies, self-efficacy

## Abstract

**Purpose:**

This study explores how mental health literacy relates to coping behavior in junior high school students, focusing on mediation of the association by self-efficacy and moderation by perceived teacher support.

**Methods:**

Information was collected from 631 junior high school students using various questionnaires, including the Adolescent Coping Strategies Scale, the Chinese version of the Adolescent Mental Health Literacy Scale, the General Self-Efficacy Scale, and the Student Perceived Teacher Support Behavior Scale.

**Results:**

All primary variables, including mental health literacy, self-efficacy, perceived teacher support, and positive coping strategies, were significantly intercorrelated. Self-efficacy was found to mediate the association between mental health literacy and positive coping strategies. Additionally, perceived teacher support moderated both the pathway from mental health literacy to self-efficacy and the subsequent development of positive coping strategies.

**Conclusion:**

Mental health literacy positively predicts the development of positive coping strategies strategies in junior high school students. Self-efficacy partially explains this relationship, indicating that mental health literacy influences coping both directly and indirectly by strengthening students' confidence in their abilities. Perceived teacher support exhibits a moderating effect that enhances the positive link between mental health literacy and self-efficacy, while attenuating the link between self-efficacy and positive coping strategies. The findings revealed the complex mediating and moderating mechanisms between mental health literacy and positive coping styles, and have profound implications for schools in enhancing positive coping styles among junior high school students.

## 1 Introduction

Positive coping strategies refer to the proactive use of cognitive and behavioral efforts to manage stress and mitigate its adverse effects. Huang et al. (2000) suggested that such strategies are typically problem-focused and involve actions like actively seeking assistance and implementing practical solutions to challenges. Junior high school corresponds to a developmental stage characterized by rapid physical maturation, psychological changes, and increasing academic demands. During this period, students frequently encounter interpersonal conflicts, academic setbacks, and psychological stressors. Without appropriate coping mechanisms, these challenges may lead to maladaptive responses, thereby worsening adjustment difficulties and negatively affecting both mental health and long-term development. Luo and Jin (2016) demonstrated that positive coping strategies strategies enable individuals to manage stress more effectively, improve problem-solving capacity, and support healthy psychological and physical development. Accordingly, the development of effective and rational coping approaches is essential for promoting wellbeing and adaptive functioning in junior high school students.

Health literacy refers to an individual's ability to understand health information and apply health knowledge based on that understanding to improve their own health. Mental health literacy, on the other hand, represents the beliefs and knowledge associated with the maintenance of mental wellbeing, recognition of mental issues and therapies, reductions in stigma, and enhancement of help-seeking efficacy (Kutcher et al., 2016). Middle school students are in a period of rapid physical and psychological development, during which mental health literacy plays an irreplaceable role in emotional regulation, stress management, and seeking psychological help. de Carvalho and da Luz Vale-Dias (2021) reported that higher mental health literacy tends to be associated with a perception of being more capable of handling life challenges and a greater likelihood of adopting constructive coping strategies. In contrast, Liao (2014) found that individuals with poor mental health literacy often display maladaptive health-related behaviors and are more prone to using dysfunctional coping responses. As a multidimensional construct involving knowledge, attitudes, and help-seeking intentions, mental health literacy is a key determinant of how individuals respond to stress. Accordingly, the first hypothesis is proposed: that mental health literacy significantly predicts positive coping strategies among junior high school students.

Self-efficacy, first introduced by Bandura (1977), is a central aspect of social cognitive theory and represents the confidence of an individual in their capacity to successfully complete particular tasks under given conditions. Social cognitive theory (Bandura, 1986) posits that individuals' knowledge base, as a cognitive resource, influences their self-evaluation and beliefs about personal capabilities, thereby driving behavioral choices. This forms a transmission mechanism in which cognitive resources affect ability beliefs, and these beliefs in turn drive performance. Schwarzer et al. (1997a) further conceptualized self-efficacy as a stable and extensive sense of confidence in how one copes with a variety of demanding situations. Wang (2002) emphasized that self-efficacy reflects an individual's perceived competence in task completion and influences behavioral choices when confronting challenges. Ai and Zhou (2018) further reported that higher stigma toward mental illness is associated with lower mental health literacy and reduced social self-efficacy. Conversely, Li (2023) reported that mental health literacy positively predicts adolescents' self-efficacy. Geng and Zhao (2022) observed positive links between self-efficacy and problem-solving abilities and positive coping strategies styles. Similarly, Knowles et al. (2020) demonstrated that self-efficacy directly shapes individuals' selection of coping strategies. As an important variable that differs among individuals, self-efficacy may serve as a psychological mechanism through which mental health literacy promotes positive coping strategies. However, research examining these relationships, particularly among junior high school students, remains limited. Therefore, the second hypothesis is proposed, stating that self-efficacy mediates the link between mental health literacy and positive coping strategies.

Perceived teacher support encompasses student perception of the academic, emotional, and interpersonal support provided by teachers within and beyond the classroom (Ouyang, 2005). It includes teachers' efforts to create supportive learning environments, facilitate academic development, and offer emotional encouragement. According to Bandura's (1986) triadic reciprocal determinism in social cognitive theory, individual development and adaptation result from the ongoing interaction among personal factors, behavior, and environmental factors. Perceived teacher support, as a key social resource at the environmental level for middle school students, influences individuals' self-efficacy and positive coping strategies. Zhu (2016) found that social support from key sources, including teachers, helps adolescents adopt more positive coping strategies strategies when facing adversity. Adolescents receiving greater social support tend to exhibit greater health literacy and a preference for positive coping strategies, and Farmanova et al. (2018) suggested that support from significant others promotes the acquisition of health-related knowledge and enhances health literacy, thereby encouraging more constructive responses to challenges. Self-determination theory (Ryan and Deci, 2000) suggests that when individuals perceive support from important others, their three basic psychological needs—autonomy, competence, and relatedness—are met. This sense of fulfillment helps transform external knowledge resources into internal beliefs about ability. Within the school context, teachers are vital for students' development. Perceived teacher support contributes to student autonomy, relatedness, and competence, thereby creating favorable conditions for translating mental health literacy into enhanced self-efficacy. When teacher support is strong, students may more readily internalize mental health knowledge as confidence in their own abilities. Huang and Liu (2025) demonstrated that perceived emotional care and inclusive attitudes from teachers can boost students' self-confidence and engagement in learning activities. Ji and Zhao (2021) further reported that teacher support behaviors enhance academic self-efficacy and promote proactive coping tendencies. Drawing on these findings, the third hypothesis is proposed: perceived teacher support moderates the association between mental health literacy and self-efficacy, as well as the link between self-efficacy and positive coping strategies among junior high school students.

In summary, this study aimed to examine the relationship between mental health literacy and positive coping styles among junior high school students, together with an investigation of the mechanisms underlying the relationship. The study first explored the ability of mental health literacy to predict positive coping styles. Second, it also analyzed the mediating effect of self-efficacy on the relationship between the two, aiming to clarify the indirect pathway. Finally, the study assessed the moderating role of perceived teacher support on the relationships between mental health literacy and self-efficacy and between self-efficacy and positive coping styles. This study constructs a moderated mediation model to explore the differential moderating mechanisms of environmental support at various stages in the process by which mental health literacy influences positive coping strategies through self-efficacy. Data were collected via questionnaires from middle school students, and mediation and moderation analyses were employed to test the hypotheses.

## 2 Research approach

### 2.1 Study participants

This study employed a combination of convenience sampling and cluster sampling to recruit participants. For the offline survey, four regular junior high schools in Heilongjiang Province and Sichuan Province were selected. With the consent of school principals, teachers, and parents, classes were randomly selected from grades 7 to 9 for cluster sampling. An anonymous paper-based questionnaire was administered; prior to distribution, students were fully informed about the research purpose, procedures, and confidentiality principles. Participation was entirely voluntary, and students had the right to decline. The online survey was conducted via an online questionnaire platform targeting junior high school students nationwide. A total of 742 questionnaires were collected. After excluding invalid responses—such as those failing lie-detection items, with excessively short completion times, or showing obvious missing data or patterned answers—631 valid questionnaires remained, yielding an effective response rate of 85.04%. This study has been reviewed and approved by the Ethics Committee of Jiamusi University (approval number: 2024-600-24). The students included 278 males (44.1%) and 353 females (55.9%). Of these, 181 participants (28.7%) were first-year students, 294 (46.4%) were second-year, and 156 (24.7%) were third-year. The majority of students (542; 85.9%) were from rural areas, while 89 students (14.1%) resided in urban settings. Additionally, 181 participants (28.7%) were only children, whereas 450 (71.3%) had siblings.

### 2.2 Research tools

#### 2.2.1 Secondary school students' Coping Strategies Scale

Coping behaviors were assessed with the Coping Strategies Scale for Middle School Students developed by Huang et al. (2000). This instrument comprises six dimensions: problem-solving, help-seeking, endurance, avoidance, emotional release, and fantasy. Among these, problem-solving and help-seeking are categorized as positive coping strategies, whereas the remaining four dimensions represent malpositive coping strategies, yielding a total of 30 items (Huang et al., 2000). This study utilized only the positive coping subscale, which includes 15 items assessed using a five-point Likert scale. Huang et al. (2000) reported the internal consistency and structural validity of the Positive Coping Scale. In this study, the Cronbach's α for the positive coping subscale was 0.875, with coefficients of 0.898 and 0.727 for its two constituent dimensions.

#### 2.2.2 Chinese version of the Adolescent Mental Health Literacy Scale

Mental health literacy was assessed using the Chinese version of the Adolescent Mental Health Literacy Scale (UMHL-A), originally established by Kågström et al. (2023) and revised by Peng and Wang (2024). The instrument includes two primary components: a knowledge subscale and an attitude subscale. The knowledge component encompasses two factors (knowledge of both general mental health and mental disorders), and contains eight items assessed on a five-point Likert scale. The attitude component includes help-seeking attitudes and stigma-related beliefs, comprising nine items assessed on a three-point Likert scale. The overall score is obtained by summing all 17 items, and higher scores reflect greater mental health literacy. The revised Chinese version of the scale has demonstrated good reliability and structural validity (Peng and Wang, 2024). In this study, the overall Cronbach's α was 0.756, with values of 0.719 and 0.738 for the knowledge and attitude subscales, respectively.

#### 2.2.3 General self-efficacy scale

Self-efficacy was evaluated using the General Self-Efficacy Scale (GSES), created by Schwarzer et al. (1997b) and revised by Wang et al. (2001). This scale includes 10 items assessed using a four-point Likert scale. The Chinese version of the scale has been proven to have good reliability and structural validity (Wang et al., 2001). In this study, the Cronbach's α was 0.897.

#### 2.2.4 Student perceived teacher support behavior scale

Perceived teacher support was evaluated with the scale developed by Ouyang (2005), which was adapted from the research on teachers' differential behaviors by Babad (1990). This instrument includes three aspects, namely, academic, emotional, and competence support, comprising 19 items overall assessed on a six-point Likert scale. Previous studies have shown that the scale has good reliability and structural validity (Wang, 2025). In this study, the overall Cronbach's α was 0.933, with subscale values of 0.873, 0.829, and 0.800, respectively.

### 2.3 Data analysis

Data were analyzed using SPSS 27.0 (International Business Machines Corporation IBM, Armonk, NY, USA). Common method bias was assessed via Harman's single-factor analysis; Spearman correlation analysis was used to examine the relationships among variables; mediation effects were tested using the PROCESS 4.2 (Dr. Andrew F. Hayes, Calgary, AB, Canada) macro, with Model 59 employed to test the moderating effect of perceived teacher support on the mediation model. All models were corrected using bootstrap resampling with 5,000 iterations; an effect was considered significant if the 95% confidence interval did not include zero.

## 3 Results

### 3.1 Assessment of common method bias

Because this study used self-report scales to collect data, which can lead to common method bias, the Harman single-factor method was used to include the mental health literacy, self-efficacy, positive coping strategies and perceived teacher support in an exploratory factor analysis. A total of 16 factors with eigenvalues greater than 1 were extracted. The first of these factors represented 23.83% of the overall variance. This value falls below the critical 40% threshold, indicating minimal common method bias in this dataset.

### 3.2 Descriptive statistics and correlations

Table 1 shows the descriptive statistics for the primary variables. The mean score for mental health literacy among junior high school students was 53.05 (SD = 5.56), exceeding the scale midpoint of 42, indicating an above-average level. The mean score for positive coping strategies was 55.10 (SD = 8.42), also higher than the midpoint of 45, suggesting a slightly above-average level of positive coping strategies. The mean self-efficacy score was 25.04 (SD = 6.14), which is approximately equal to the scale midpoint of 25, reflecting a moderate level. Perceived teacher support had a mean score of 82.39 (SD = 16.03), exceeding the midpoint of 66.5, indicative of relatively high levels of perceived support.

**Table 1:** Descriptive statistics and correlations for study variables (*N* = 631).

Research variables	*M*	SD	1	2	3	4
1 Mental health literacy	53.05	5.56	1			
2 Positive coping strategies	55.10	8.42	0.538^**^	1		
3 Self-efficacy	25.04	6.14	0.443^**^	0.636^**^	1	
4 Perceived teacher support	82.39	16.03	0.347^**^	0.581^**^	0.537^**^	1

^**^p < 0.01.

Pearson correlation analyses revealed significant positive associations among all study variables. As illustrated in Table 1, mental health literacy showed positive correlations with positive coping strategies (*r* = 0.538, *p* < 0.01), self-efficacy (*r* = 0.443, *p* < 0.01), and perceived teacher support (*r* = 0.347, *p* < 0.01). Positive coping strategies were also significantly correlated with self-efficacy (*r* = 0.636, *p* < 0.01) and perceived teacher support (*r* = 0.581, *p* < 0.01). Furthermore, self-efficacy was positively associated with perceived teacher support (*r* = 0.537, *p* < 0.01). These results support the hypothesized relationships among the variables.

### 3.3 The mediating influence of self-efficacy on the association between mental health literacy and coping strategies

The above analysis showed that there was a significant correlation between the variables and that collinearity may exist. Therefore, before testing the effect, the predictive variables in the equation were standardized, and collinearity diagnostics were performed. The results showed that the variance inflation factors VIF (1.271, 1.571, and 1.436) of all of the predictors were less than five. Therefore, there was no serious collinearity in the data used for this study, indicating that they were suitable for further mediation effect tests and moderating effect.

Self-efficacy mediation of the link between mental health literacy and coping strategies was examined with the PROCESS macro (Model 4) produced by Hayes. As shown in Table 2, mental health literacy significantly predicted positive coping strategies (β = 0.538, *p* < 0.001). The inclusion of self-efficacy as a mediating factor did not affect the significance of the relationship, although the coefficient was reduced (β = 0.318, *p* < 0.001), indicative of partial mediation. Further analyses showed that mental health literacy was predictive of self-efficacy (β = 0.443, *p* < 0.001), and self-efficacy was predictive of positive coping strategies (β = 0.495, *p* < 0.001).

**Table 2:** Regression results of the mediating influence of self-efficacy on the association between mental health literacy and positive coping strategies.

Dependent variables	Predictor variables	*R* ^2^	*F*	β	*SE*	*t*	95% CI
Self-efficacy	Mental health literacy	0.196	153.601	0.443	0.040	12.394^***^	(0.412, 0.567)
Positive coping strategies	Mental health literacy	0.289	255.636	0.538	0.051	15.989^***^	(0.714, 0.914)
Positive coping strategies	Mental health literacy	0.486	297.091	0.318	0.048	9.971^***^	(0.387, 0.577)
	Self-efficacy			0.495	0.044	15.524^***^	(0.593, 0.765)

^***^p < 0.001.

**Figure 1:**
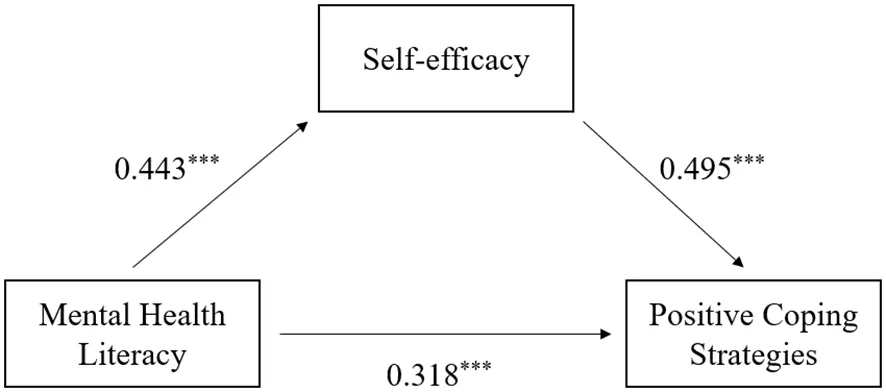
Mediation model illustrating the role of self-efficacy between mental health literacy and positive coping strategies. ^***^p < 0.001.

The direct effect of mental health literacy on positive coping strategies was estimated at 0.318, with a 95% CI of (0.256, 0.381). The exclusion of zero indicates a marked direct influence. In addition, the indirect effect of mental health literacy on positive coping strategies through self-efficacy was 0.219 (95% CI: 0.179, 0.262), also not including zero. These results demonstrate that self-efficacy is a partial mediator of the link between mental health literacy and positive coping strategies, thereby providing support for the mediation model. Results are detailed in Table 3 and Figure 1.

**Table 3:** Mediation analysis of self-efficacy in the link between mental health literacy and positive coping strategies in junior high school students.

Effect	β	SE	95% CI	Effect size (%)
Direct effect	0.318	0.032	(0.256, 0.381)	59.18
Indirect effect	0.219	0.021	(0.179, 0.262)	40.82
Total effect	0.537	0.034	(0.472, 0.604)	

### 3.4 Perceived teacher support as a moderator of the relationship between mental health literacy and positive coping strategies

Building on the mediation findings, we further investigated whether perceived teacher support moderates the relationships among mental health literacy, self-efficacy, and positive coping strategies. To explore these relationships, moderated mediation was assessed using PROCESS 4.2 (Model 59) with 95% CIs.

**Figure 2:**
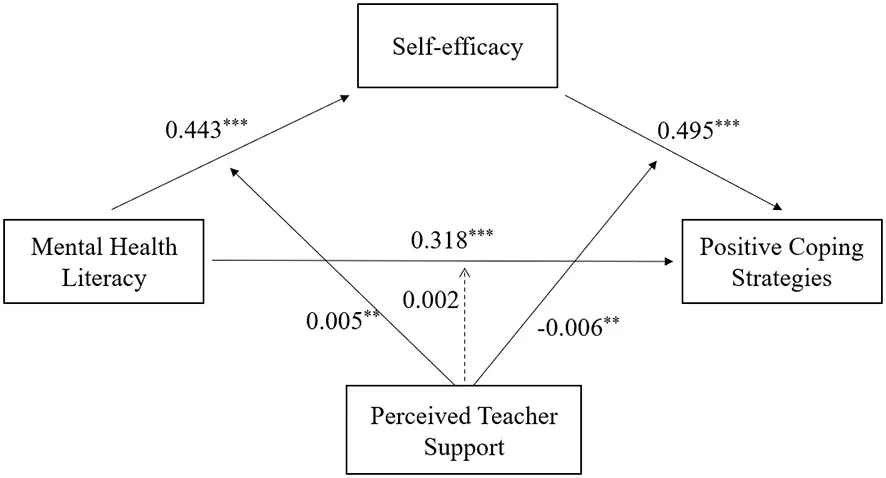
Moderated mediation model of the links between mental health literacy and positive coping strategies in junior high school students. ^**^p < 0.01. and ^***^p < 0.001.

As shown in Table 4 and Figure 2, both mental health literacy and perceived teacher support were significantly predictive of self-efficacy [β = 0.005, *p* < 0.01, 95% CI = (0.001, 0.009)], indicating that perceived teacher support strengthens the positive association between mental health literacy and self-efficacy. In contrast, the interaction between mental health literacy and perceived teacher support showed no marked prediction of positive coping strategies [β = 0.002, *p* = 0.522, 95% CI = (−0.003, 0.006)], suggesting that perceived teacher support does not moderate this direct relationship. However, the link between self-efficacy and perceived teacher support was significantly predictive of positive coping strategies [β = −0.006, *p* < 0.01, 95% CI = (−0.010, −0.002)]. This indicates that perceived teacher support negatively moderates the relationship between self-efficacy and positive coping strategies.

**Table 4:** Moderating influence of perceived teacher support on the associations among mental health literacy, self-efficacy, and positive coping strategies.

Predictor	Self-efficacy				Positive coping strategies			
	β	*t*	SE	95% CI	β	*t*	SE	95% CI
Mental health literacy	0.336	8.915^***^	0.038	(0.262, 0.410)	0.435	9.321^***^	0.047	(0.343, 0.526)
perceived teacher support	0.165	12.715^***^	0.013	(0.139, 0.190)	0.149	8.812^***^	0.017	(0.116, 0.183)
Self-efficacy					0.490	10.584^***^	0.046	(0.399, 0.581)
Mental health literacy × perceived teacher support	0.005	2.666^**^	0.002	(0.001, 0.009)	0.002	0.641	0.002	(−0.003, 0.006)
Self-efficacy × perceived teacher support					−0.006	−2.736^**^	0.002	(−0.010, −0.002)
*R* ^2^	0.371				0.551			
*F*	123.107^***^				153.659^***^			

All continuous variables were standardized prior to regression analysis.

^**^p < 0.01.

^***^p < 0.001.

**Figure 3:**
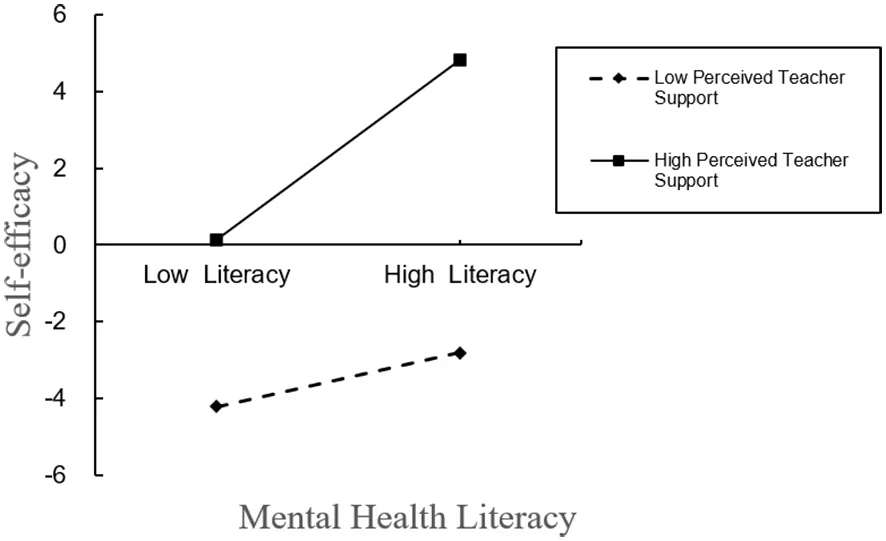
Simple slope analysis of the moderating influence of perceived teacher support on the link between mental health literacy and self-efficacy.

To further clarify the moderating influence of perceived teacher support on the link between mental health literacy and self-efficacy, a simple slope analysis was conducted at one standard deviation above and below the mean level of perceived teacher support. As illustrated in Figure 3, both low and high levels of perceived teacher support substantially influenced the link between mental health literacy and self-efficacy (low: β = 0.251, *t* = 5.464, *p* < 0.001; high: β = 0.421, *t* = 8.013, *p* < 0.001). The significance of the interaction (β = 0.005, *p* < 0.01) further confirms a positive moderating effect. Specifically, greater perceived teacher support amplifies the positive impact of mental health literacy on self-efficacy. As shown in Figure 3, the slope for students reporting high teacher support is steeper than that for those with low support, indicating a stronger increase in self-efficacy as mental health literacy improves. Conversely, among students perceiving lower teacher support, the increase in self-efficacy associated with higher mental health literacy is more gradual. Together, these results demonstrate that the level of perceived teacher support positively moderates the link between mental health literacy and self-efficacy in students in junior high school.

**Figure 4:**
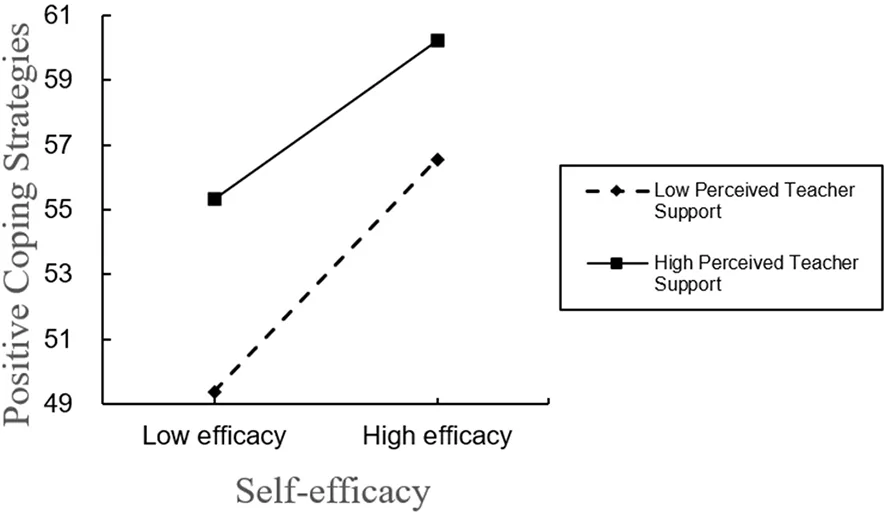
Simple slope analysis of the moderating influence of perceived teacher support on the link between self-efficacy and positive coping strategies.

A similar simple slope analysis was performed to examine the moderating influence of perceived teacher support in the link between self-efficacy and positive coping strategies one standard deviation above and below the mean level of perceived teacher support (Figure 4). Both low and high perceived teacher support markedly influenced this relationship (low: β = 0.583, *t* = 10.072, *p* < 0.001; high: β = 0.398, *t* = 6.997, *p* < 0.001). The combined effect was significant (β = −0.006, *p* < 0.01), indicating a negative moderating role. More specifically, as perceived teacher support increases, the positive link between self-efficacy and positive coping strategies weakens. This pattern is reflected in Figure 4, where the slope corresponding to high perceived teacher support is flatter compared to that of low perceived teacher support. In practical terms, students with lower perceived teacher support show a stronger increase in positive coping behaviors as self-efficacy rises, whereas for those with higher perceived support, the increase in coping strategies is less pronounced with increasing self-efficacy. Overall, these findings indicate that perceived teacher support weakens the predictive influence of self-efficacy on positive coping strategies through its negative moderating effect among students in junior high school.

## 4 Discussion

### 4.1 Association between mental health literacy and coping strategies

Correlation analyses indicated a robust positive link between mental health literacy and the use of positive coping strategies strategies, thereby supporting the first hypothesis. In practical terms, students with greater mental health literacy were more inclined to apply constructive approaches when confronted with stressors or adverse situations. This pattern is consistent with the findings reported by Peng (2025), which indicate that enhanced mental health literacy can increase the likelihood of positive coping by strengthening individuals' understanding of psychological processes, refining coping skills, and facilitating adaptive adjustment outcomes. Similarly, Li (2018) found that middle school students possessing greater mental health knowledge are more likely to employ constructive coping mechanisms when dealing with challenges. According to the stress and coping theory (Lazarus and Folkman, 1984), coping strategies depend on individuals' cognitive appraisals of stressful situations. Adolescents with higher mental health literacy are better able to accurately identify their emotional states, form more positive cognitive evaluations, and are thus more likely to adopt adaptive coping strategies. Jorm (2012) further emphasized that mental health literacy not only shapes the capacity to identify and manage psychological difficulties but is also closely linked to health-related behaviors and coping patterns. The present findings extend this evidence to junior high school populations, demonstrating that mental health literacy is a marked positive predictor of positive coping strategies. From an applied perspective, the influence of mental health literacy appears to operate through both knowledge acquisition and attitudinal change. Accordingly, educational systems and broader social institutions should prioritize mental health education, enable access to psychological support, and foster more informed and constructive attitudes toward mental health among adolescents.

### 4.2 The mediating influence of self-efficacy

The mediation analysis indicated that self-efficacy partially mediates the link between mental health literacy and positive coping strategies, thereby supporting the second hypothesis. Specifically, mental health literacy exerted both a direct influence on coping behaviors and an indirect effect through its enhancement of self-efficacy. This fits with social cognitive theory, in which Bandura (1977) observed that self-efficacy was a central determinant of behavioral selection and persistence. Students with higher mental health literacy typically possess a more comprehensive understanding of psychological concepts and are better able to apply this knowledge to interpret and manage emotional distress. These successful applications reinforce their confidence in handling difficulties, thereby strengthening self-efficacy. Supporting this mechanism, (Schwarzer and Renner 2000) reported that greater self-efficacy led to the perception of stress as manageable and are therefore more inclined to adopt proactive coping strategies. According to social cognitive theory, knowledge about mental health serves as a cognitive resource that influences individuals' perceptions of their capabilities and self-confidence, thereby regulating behavioral orientation through belief systems. Liu et al. (2015) found that there is a significant correlation between self-efficacy and coping styles, with self-efficacy significantly predicting positive coping strategies in a positive direction. In this context, mental health literacy contributes both cognitive resources and practical skills, while self-efficacy provides the motivational foundation necessary to translate these resources into action. Students with stronger mental health literacy not only understand how to regulate emotions and address stress but also develop confidence in their capacity to do so. This confidence, in turn, promotes the consistent use of positive coping strategies among students.

Importantly, these findings suggest that the pathway from mental health literacy to coping behavior is not purely direct but operates through a more complex mechanism involving individual belief systems. Mental health literacy supplies the informational and attitudinal basis, whereas self-efficacy enables the effective application of this knowledge in real-world contexts. The interaction of these factors facilitates the development and maintenance of positive coping strategies patterns.

### 4.3 The moderating influence of perceived teacher support

The moderation analyses demonstrated that junior high school students' perceptions of teacher support contribute to the relationships among mental health literacy, self-efficacy, and positive coping. Perceived teacher support strengthened the positive link between mental health literacy and self-efficacy, while attenuating the positive relationship between self-efficacy and positive coping strategies behaviors, thereby confirming the third hypothesis. In practical terms, greater perceived teacher support intensified the extent to which mental health literacy predicted self-efficacy. Conversely, greater perceived support corresponded to a reduced influence of self-efficacy on positive coping strategies. These results highlight the nuanced moderating function of teacher support in the pathway from mental health literacy to coping, offering a more refined understanding of the interplay among these constructs. The research findings support the perspective of social cognitive theory, demonstrating a dynamic interplay among individual, behavioral, and environmental factors. Psychological wellbeing literacy and self-efficacy in middle school students represent individual cognitive factors, while positive coping strategies serve as behavioral factors. Perceived teacher support, as a key social resource within the environmental context, dynamically moderates the influence of students' cognitive resources on adaptive behaviors.

First, the findings demonstrate that perceived teacher support significantly conditions how health literacy is linked to self-efficacy. The strength of this relationship depends on the degree of perceived support from the teachers. At higher levels of support, mental health literacy more strongly predicts self-efficacy, while low support weakens the relationship. Social cognitive theory posits that self-efficacy is shaped not only by direct mastery experiences and observational learning but also by social persuasion and affective states. Bandura (2001) argues that efficacy beliefs are cultivated through experience, modeling, and encouragement. In school contexts, teachers are central providers of such encouragement, helping students establish realistic goals, offering constructive feedback, and recognizing accomplishments. Empirical evidence supports this view: Liu (2020) found that teacher support significantly improves middle school students' self-efficacy, with benefits observed in both academic outcomes and psychological adjustment. As salient figures in adolescents' daily school experiences, teachers contribute to students' development through instructional guidance, emotional support, and affirmation of competence. These forms of support function as powerful sources of social persuasion, enabling students to translate mental health knowledge into a sense of confidence in their coping abilities. When students perceive strong teacher support, they tend to treat their mental health knowledge as a dependable resource, thereby strengthening their confidence in managing challenges. This moderating effect suggests that teacher support, as an external contextual resource, amplifies the influence of mental health literacy, as an internal cognitive asset, on self-efficacy. In supportive classroom environments characterized by emotional warmth, academic guidance, and validation of competence, students are provided with a secure psychological space. Such conditions facilitate the application of mental health knowledge to real-world situations and reinforce students' beliefs in their capabilities through positive teacher feedback.

Second, the results indicate that perceived teacher support also shows a marked negative moderating influence on the link between self-efficacy and positive coping strategies. As perceived teacher support increases, the impact of self-efficacy on positive coping strategies diminishes. Specifically, when perceived support is low, self-efficacy has a stronger positive association with coping strategies, whereas when perceived support is high, this association becomes weaker. As an external contextual factor, teacher support can shape how self-efficacy translates into behavior. This pattern is consistent with self-determination theory, as Ryan and Deci (2000) proposed that supportive environments fulfill individuals' needs for autonomy, relatedness, and competence, thereby promoting both motivation and engagement. Evidence from (Ji and Zhao 2021) further suggests that teacher support can indirectly enhance academic outcomes by increasing students' self-efficacy, which in turn encourages more proactive problem-solving when difficulties arise. The findings of this study support the perspective of self-determination theory: students who perceive higher levels of teacher support are more likely to attribute their success to teachers' assistance. With access to strategic guidance, emotional comfort, and practical help from teachers whenever needed, these students tend to engage in more diligent learning behaviors. This suggests that coping styles do not solely depend on individual students' confidence in their own abilities, but are significantly influenced by external support. In contrast, students who perceive lower levels of teacher support, lacking such external environmental support, may rely more heavily on their own confidence as a key motivator for effortful learning when facing stress. Consequently, for students with low perceived teacher support, their sense of self-efficacy plays an enhanced role in promoting positive coping strategies. The observed moderating pattern indicates that the indirect pathway linking mental health literacy to coping through self-efficacy is contingent on the level of perceived teacher support. In the first stage of this process, teacher support strengthens the association between mental health literacy and self-efficacy, while in the subsequent stage, it weakens the dependence of coping behaviors on self-efficacy, emphasizing that development arises from dynamic interactions between individual characteristics and environmental influences. From a practical standpoint, the results underscore the importance of fostering supportive teacher-student relationships in mental health education. Efforts to enhance students' mental health literacy and self-efficacy should be accompanied by the cultivation of a supportive educational climate, as this combination is likely to maximize the effectiveness of interventions aimed at promoting positive coping strategies.

## 5 Conclusion

The results of this study confirmed that the mental health literacy of junior high school students is significantly and positively predictive of positive coping styles. Self-efficacy plays a partial mediating role between mental health literacy and positive coping styles, indicating that mental health literacy has both a direct effect on positive coping styles and an indirect influence through the enhancement of students' self-efficacy. Perceived teacher support plays a positive moderating role between mental health literacy and self-efficacy, and a negative moderating role between self-efficacy and positive coping styles. These findings will aid the development of intervention programs to enhance positive coping styles among junior high school students, including improvements in teacher support for students in the academic, emotional, and competence domains.

## 6 Limitations and future directions

However, this study still has some limitations. First, the cross-sectional design may limit the generalizability of the findings; future longitudinal studies could be conducted to explore the developmental patterns of the variables and their dynamic relationships. Second, this study only examined the relationships among mental health literacy, self-efficacy, perceived teacher support, and positive coping strategies. Further in-depth research is needed to validate and refine the proposed model. Future studies could further investigate individual factors such as psychological resilience and emotional regulation, as well as environmental factors like family and peer support, to jointly explore the mechanisms underlying the development of adolescents' positive coping strategies, thereby providing more robust theoretical foundations and empirical evidence for school-based mental health education practices.

## Data Availability

The raw data supporting the conclusions of this article will be made available by the authors, without undue reservation.
